# Adrenal myelolipoma: from tumorigenesis to management

**DOI:** 10.11604/pamj.2019.34.180.20891

**Published:** 2019-12-05

**Authors:** Wassim Alaoui Mhammedi, Hicham Ouslim, Abdelghani Ouraghi, Mohammed Irzi, Amine Elhoumaidi, Amine Elhoumaidi, Mehdi Chennoufi, Mohammed Mokhtari, Anouar Elmouden, Ali Barki

**Affiliations:** 1 Urology Department, Mohammed the Sixth University Hospital, Oujda, Morocco

**Keywords:** Adrenal myelolipomas, incidentaloma, conservative management, laparoscopic adrenalectomy

## Abstract

Adrenal myelolipoma (MLS) is a rare, benign and non-functional neoplasm, composed of adipose tissue and myeloid. We report a rare case of adrenal myelolipoma of a 20-year-old female revealed with chronic abdominal pain. Computed tomography (CT) scan of the abdomen guided diagnosis and surgical resection was performed given symptomatic and bulky mass. Histological examination confirmed the diagnosis. At 18 months after the surgery, the patient had no evidence of recurrence. The diagnosis of MLS is radiological. Therapeutic abstention is the rule for a small, asymptomatic tumor. The surgical removal is indicated when it is bulky (exceeds 7cm), symptomatic or hormonal activity.

## Introduction

Adrenal myelolipoma (MLS) is a rare, benign, non-functional tumor. Composed of mature adipose tissue and hematopoietic elements, the MLS are often a fortuitous discovery and detected during imaging procedures. Usually respected, surgical removal is indicated when tumor is bulky, compressive or at risk of haemorrhage. We report a case of adrenal myelolipoma and review of the literature.

## Patient and observation

**Figure 1:**
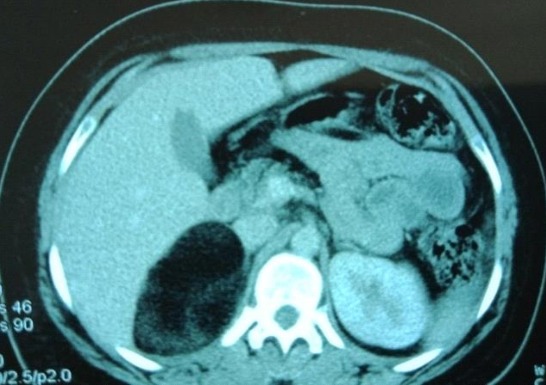
CT scan image showing heterogenous fatty masses 8.35 × 5.73cm with negative attenuation value (-112 HU) in a right adrenal mass

**Figure 2:**
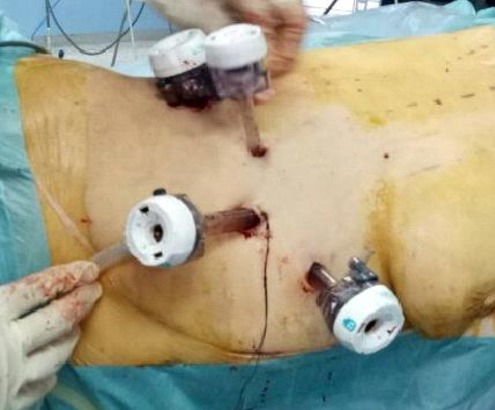
Trocar positioning

**Figure 3:**
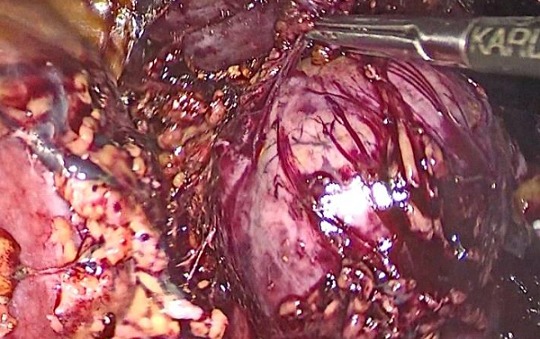
Laparoscopic adrenalectomy

**Figure 4:**
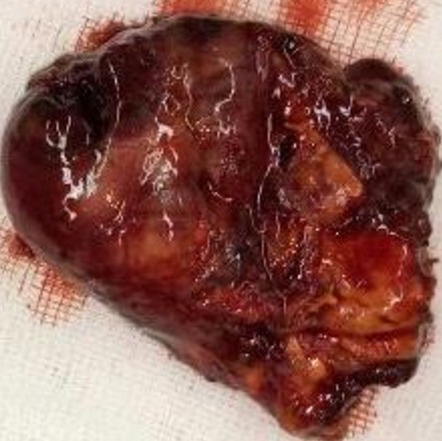
Right adrenalectomy specimen

**Figure 5:**
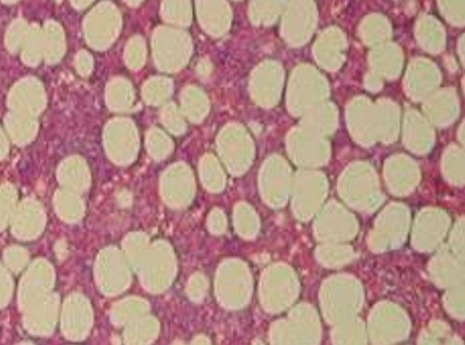
Histopathology showing mature fat cells with hematopoitic cells typical of adrenal myelolipomas

A 20-year-old female presented with episodic abdominal pain for 6 months. Her medical history was notable for diabetes. On physical examination, there was no significant finding, with a BMI of 29 kg/m^2^. An ultrasonogram (US) revealed adrenal masses. Computed tomography (CT) scan showed an 8.35 × 5.73cm right adrenal mass, composed of fat tissue, which has a -112 HU value (Figure 1). Laboratory investigations revealed the non-functioning nature of the adrenal mass, were also within normal limits: normetanephrine: 0.43μmol/24h (reference range 0.40-2.10μmol/24h). Metanephrine: <0.10μmol/24h (reference range 0.00-0.49μmol/24h). Serum cortisol 8 h: 11.7μg/dl (normal; 7-28μg/dl) 24-hour urine cortisol excretion 36 μg/24h (normal; 700-1500 μg/24h). A surgical right adrenalectomy was performed through laparoscopic transperitoneal approach (Figure 2 and Figure 3). Histopathology revealed a large and encapsulated mass measuring 8.5cm × 8.5cm × 4.5cm (Figure 4). Microscopy revealed a lesion was composed of mature adipose tissue with hematopoietic cells without signs of cell atypia, thus confirming the diagnosis of adrenal myelolipoma (Figure 5). At 18 months after the surgery, the patient had no evidence of recurrence.

## Discussion

Adrenal myelolipomas are benign tumours with a reported incidence of 2% to 4% of all adrenal tumours. The tumour was initially described by Giercke in 1905 and later the term “myelolipoma” was coined by Oberling in 1929 [1]. They are composed of variable proportions of mature adipose tissue and active hematopoietic elements. They are usually discovered incidentally on autopsy, surgery, or as an incidentalomas on imaging for other reasons [2]. Adrenal myelolipoma (AML) is the second most common primary adrenal incidentaloma representing 15% of adrenal incidentalomas. Myelolipomas can rarely appear at other sites of the human body, as well, hence the term extra-adrenal myelolipoma [3]. Adrenal myelolipoma is mostly a unilateral tumor without symptoms of endocrine disorder, with bilateral masses occurring in about 10% of cases [4]. Tumour size has been reported in literature from a few millimetres to more than 30cm, but rarely exceeds 5cm [2]. They frequently present between the fifth and seventh decades of life without a predominance in either sex, though there is a greater incidence in the right adrenal gland [5]. The youngest patient was 1-year-old at the diagnosis, while the oldest was 83 years old [3]. More than 400 cases were reported in the literature.

The hypothesis of the tumorigenesis: the fat components are derived by the mesenchymal stem cells of stromal fat of adrenal cortex under certain stimuli. Mature adipocytes begin to accumulate and become inflammatory stimulating neighbouring adrenal cortex tissue. During the tumour growth, haematopoietic cells in the central part fat until they are fully differentiated and division stops [6]. Most myelolipomas are asymptomatic and discovered incidentally on abdominal imaging. Some may present with abdominal pain because of huge size or hemorrhage or necrosis within tumor [7]. MLS is rarely manifested by spontaneous retroperitoneal hemorrhage, which remains the most serious complication giving up to 80% of deaths [8]. Although the tumor itself is non-functioning, there is 10% incidence of associated endocrine disorders such as Cushing's disease, Conn's syndrome, diabetes and congenital adrenal hyperplasia. Besides, rare associations with sickle cell anemia and thalassemia have been reported [9].

The issue on the necessity of hormonal evaluation of AML is debated. In the American Association of Clinical Endocrinologists (AACE) guideline, Zeiger *et al*. did not propose hormonal evaluation for obvious myelolipoma [10]. The clinical picture should be the major factor in determining the necessity for hormonal work-up [3]. It is very important to differentiate between myelolipomas and other lipomatous adrenal tumors. These masses include adrenocortical adenoma, adrenocortical carcinoma, retroperitoneal liposarcoma, exophytic renal angiomyolipoma, and adrenal lipoma [3]. Ultrasound of the abdomen can differentiate the supra-renal mass from the kidneys, but it cannot confirm a myelolipoma [2]. On CT scans, myelolipomas are well-circumscribed, round or elliptical, hypodense and heterogenous masses. Attenuation values of -120 to -90 HU are characteristic of the adipose tissue, and the presence of fat density is useful and pivotal in diagnosing the mass as a myelolipoma [3]. MRI allows to determine the various structural components of myelolipomas and therefore appears to be a very reliable technique in the diagnosis and characterization of this rare adrenal pathology. Three different morphologies on MRI when imaging myelolipomas: (a) homogenous, hyperintense masses on T1 weighted sequences with intermediate signals on T2 weighted images, suggestive for predominantly fat-containing lesions; (b) heterogenous masses with fat intensity areas and hyperintense areas on T2 weighted images and on post-contrast T1-weighted sequences, suitable for mixed fatty and myeloid elements; (c) nodules hypointense on T1 weighted sequences and hyperintense on T2-weighted images and after gadolinium administration suggesting tumors of myeloid elements [11].

Imaging studies are accurate in diagnosing AML in up to 90% of the cases [12]. If CT shows non-homogenous characteristics or if the diagnosis is in doubt, an image guided needle biopsy could be performed to confirm the diagnosis, but this approach bears the risk of rupture and bleeding [2]. The surgical removal is indicated for symptomatic tumor, size greater than 7cm, metabolically active tumor and suspicion of malignancy on imaging study [12]. The laparotomy or laparoscopy can be performed. Laparoscopic adrenalectomy is safe and effective [3]. Two approaches are applied at laparoscopic surgery, the transperitoneal and retroperitoneal approach. Large tumors can be removed much more easily by the transperitoneal (TP) approach than by retroperitoneal (RP) approach. But one approach (TP or RP) over the other also does not lead to the substantial benefits either to the patients or to the surgeon [13]. All asymptomatic tumors less than 7cm in diameter were managed conservatively. The timing of follow-up was not always declared by the authors. Usually the follow-up plan consists in US or CT scan or MRI after 12 and 24 months, but there is no uniformity [14].

## Conclusion

Adrenal myelolipomas are uncommon benign tumors, most often asymptomatic and discovered incidentally in imaging diagnostics. Computed tomography can yield the diagnosis. Smaller, asymptomatic myelolipomas can be observed expectantly. The bulky (greater than 7cm), symptomatic, metabolically active tumor scan recommend surgical resection. Mini-invasive techniques performed by experienced laparoscopic surgeons are an effective and feasible way for their removal.

## Competing interests

The authors declare no competing interests.
